# Chemoradioimmunotherapy versus chemoimmunotherapy or chemoradiotherapy in extensive‐stage small‐cell lung cancer: A retrospective analysis of survival outcomes

**DOI:** 10.1002/pro6.70043

**Published:** 2025-12-22

**Authors:** Jinmin Han, Ying Hua, Xue Wu, Xiufeng Tang, Chengxin Liu

**Affiliations:** ^1^ Department of Radiotherapy Yantai Yuhuangding Hospital Affiliated to Qingdao University Yantai Shandong China; ^2^ Department of Medical Oncology Shandong Cancer Hospital and Institute Shandong First Medical University and Shandong Academy of Medical Sciences Jinan Shandong China; ^3^ Department of Internal Medicine Ziliujing District People's Hospital Zigong Sichuan China; ^4^ Department of Pharmacy Shandong Cancer Hospital and Institute Shandong First Medical University and Shandong Academy of Medical Sciences Jinan Shandong China; ^5^ Department of Radiation Oncology Shandong Cancer Hospital and Institute Shandong First Medical University and Shandong Academy of Medical Sciences Jinan Shandong China

**Keywords:** chemoimmunotherapy, chemoradioimmunotherapy, chemoradiotherapy, extensive‐stage small cell lung cancer

## Abstract

**Background and purpose:**

Current evidence is insufficient to define the value of thoracic radiotherapy (TRT) following chemoimmunotherapy (CT‐IO) in extensive‐stage small‐cell lung cancer (ES‐SCLC). We aimed to ascertain whether incorporating immunotherapy (IO) could improve survival and explore the efficacy of TRT in combination with CT‐IO among patients with ES‐SCLC.

**Methods:**

Clinical data were retrospectively analyzed. Patients were classified into two groups: IO and chemoradiotherapy (CRT). Within the IO group, we further defined two subgroups: CT‐IO and chemoradioimmunotherapy (CRT‐IO) groups.

**Results:**

A total of 206 patients were enrolled in this study. The median overall survival was 22.2 months in the CRT‐IO group, which was longer than the 16.0 months observed in the CT‐IO group (*P* = 0.002) and 19.0 months noted in the CRT group (*P* = 0.208). The objective response rate (ORR) in the CRT‐IO group (69.8%) was better than that in the CT‐IO (68.9 %, *P* = 0.929) and CRT (59.3 %, *P* = 0.227) groups.

**Conclusions:**

Considering the trend toward prolonged survival and a higher ORR in the CRT‐IO group, TRT may be feasible in IO era. Considering the economic factors and physical conditions, CRT may be an option for patients with ES‐SCLC.

## Graphical abstract

This study assessed the impact of IO and the role of TRT in ES‐SCLC treatment.The CRT‐IO group had longer mOS than the CT‐IO and CRT group.TRT may be beneficial in the IO era, CRT may be also a viable option considering economic and physical factors.

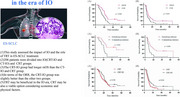

## 1 INTRODUCTION

Currently, approximately two‐thirds of patients diagnosed with small cell lung cancer (SCLC) present with extensive‐stage (ES) disease at their initial visit.^1^, ^2^ For three decades, patients with metastasis have typically received platinum‐based chemotherapy (CHT) as the standard regimen; however, their survival remains poor.^3^ Several clinical studies have indicated that immunotherapy (IO) can remarkably extend overall survival (OS) and progression‐free survival (PFS) in patients with ES‐SCLC.^4^, ^5^, ^6^, ^7^, ^8^ The IMpower133 and CASPIAN studies provided strong evidence that IO can be effectively combined with standard CHT.^4^, ^5^, ^6^ The CAPSTONE‐1 and ASTRUM‐005 studies have verified that the introduction of IO could result in favorable patient outcomes.^7^, ^8^ IO combined with CHT without thoracic radiation therapy (TRT) has been approved as a first‐line treatment for patients.^4^, ^5^, ^6^, ^7^, ^8^ Although IO has revolutionized treatment strategies, the survival benefit remains unsatisfactory, and still challenges in its application remain.

Several studies, including the Jeremic, CREST, and RTOG 0937 studies, have demonstrated that TRT can increase survival in patients with ES‐SCLC; however, it has not been uniformly adopted worldwide.^9^, ^10^, ^11^ Despite evidence confirming the efficacy of IO‐ and TRT‐alone, the benefits of TRT following IO remain unknown. TRT combined with IO has been shown to have survival benefits in patients diagnosed with non‐small cell lung cancer (NSCLC), suggesting a possible synergistic effect.^12^, ^13^, ^14^ The synergistic mechanism lies in TRT‐induced immunomodulation (release of tumor antigens and activation of immune cells) and immunotherapy‐enhanced radiotherapy efficacy (reversal of immune suppression and T‐cell infiltration).^15^, ^16^ Furthermore, a phase I trial by Welsh et al. evaluated whether the addition of TRT to IO could provide advantages for ES‐SCLC.^17^ Whether combination therapy is a promising approach is worth exploring. Therefore, we designed our study to ascertain whether integrating IO could improve survival and to explore the efficacy of TRT when combined with chemoimmunotherapy (CT‐IO) by comparing the clinical outcomes of patients with ES‐SCLC receiving chemoradioimmunotherapy (CRT‐IO), CT‐IO, or chemoradiotherapy (CRT).

## 2 MATERIALS AND METHODS

### 2.1 Patient selection and study design

Retrospective data were collected from patients with ES‐SCLC who underwent CHT or CRT between January 2016 and January 2018 and received IO between January 2019 and February 2020 at the Shandong Cancer Hospital and Institute. Eligible patients had their SCLC diagnosis made by histology or cytology, with a Karnofsky performance status (KPS) score ≥70 and adequate imaging data confirming distant metastasis. Patients with autoimmune disorders, interstitial lung disease, or other serious chronic diseases were excluded from the study. Patients who were lost to follow‐up underwent fewer than two cycles of treatment, received only CHT, and received IO in the second‐line setting. Patients were subsequently categorized into two groups: IO and CRT. Within the IO group, we further defined two subgroups: CRT‐IO and CT‐IO. Data extracted from electronic medical records included age at diagnosis, sex, KPS score, treatment details, metastatic location, date of progression or death, and the last follow‐up. Our study was approved by the Ethics Committee of the Shandong Cancer Hospital and Institute (approval number: SDTHEC202501068). Given the retrospective nature of our study, the requirement for informed consent was waived.

### 2.2 Treatment protocol and follow‐up

Intensity‐modulated radiotherapy was used to deliver the TRT. To define the gross tumor volume (GTV), contouring was performed to incorporate the residual primary tumor after treatment and regions with positive lymph nodes. Following GTV delineation, the clinical target volume (CTV) was established by expanding the GTV to cover the lymph node drainage areas at a high risk of tumor involvement. The planning target volume was created by applying a 0.5–1.0 cm expansion margin to the CTV. TRT was administered in two common regimens: 45 Gy in 30 fractions, with 1.5 Gy per fraction two times daily, or 30–60 Gy in 10–30 fractions, with 2–3 Gy per fraction once daily. The TRT dose and fractionation schedule were determined by the radiation oncologist, considering the tumor volume and performance status, as no uniform recommendation existed in the guidelines. The choice of immune agents, including pembrolizumab, nivolumab, and atezolizumab, has been inconsistent across studies. The duration of IO maintenance for patients lasts for 1–2 years or is discontinued upon disease progression or intolerable treatment‐related adverse events. The chemotherapeutic regimens used were mainly platinum‐based, with etoposide and platinum (EP) or etoposide and carboplatin (EC) being the most common combinations.

Survival data, including PFS and OS, were obtained until October 31, 2019, for the CRT group and July 31, 2024, for the IO group, respectively. OS duration was measured from the initiation of treatment to either the final follow‐up visit or the date of death. For PFS, the time interval from treatment initiation to disease progression, the most recent follow‐up, or patient death was assessed.

### 2.3 Statistical analysis

Pairwise comparisons of categorical variables between groups were conducted using chi‐square or Fisher's exact tests. Meanwhile, Kaplan–Meier method combined with the log‐rank test was used for survival analysis. Survival curves were plotted using GraphPad Prism 8.0 (GraphPad Prism Software Inc.,San Diego, CA, USA). Univariate and multivariate Cox proportional hazards regression models were used to identify independent risk factors. The latest version of the CTCAE classification was used to grade adverse events. Tumor response was assessed according to RECIST version 1.1. All statistical analyses were performed using IBM SPSS Statistics 24.0 (IBM Corp., Armonk, NY, USA). Statistical significance was set at *P*<0.05. significant.

## 3 RESULTS

### 3.1 Study flow diagram and patient baseline characteristics

**FIGURE 1:**
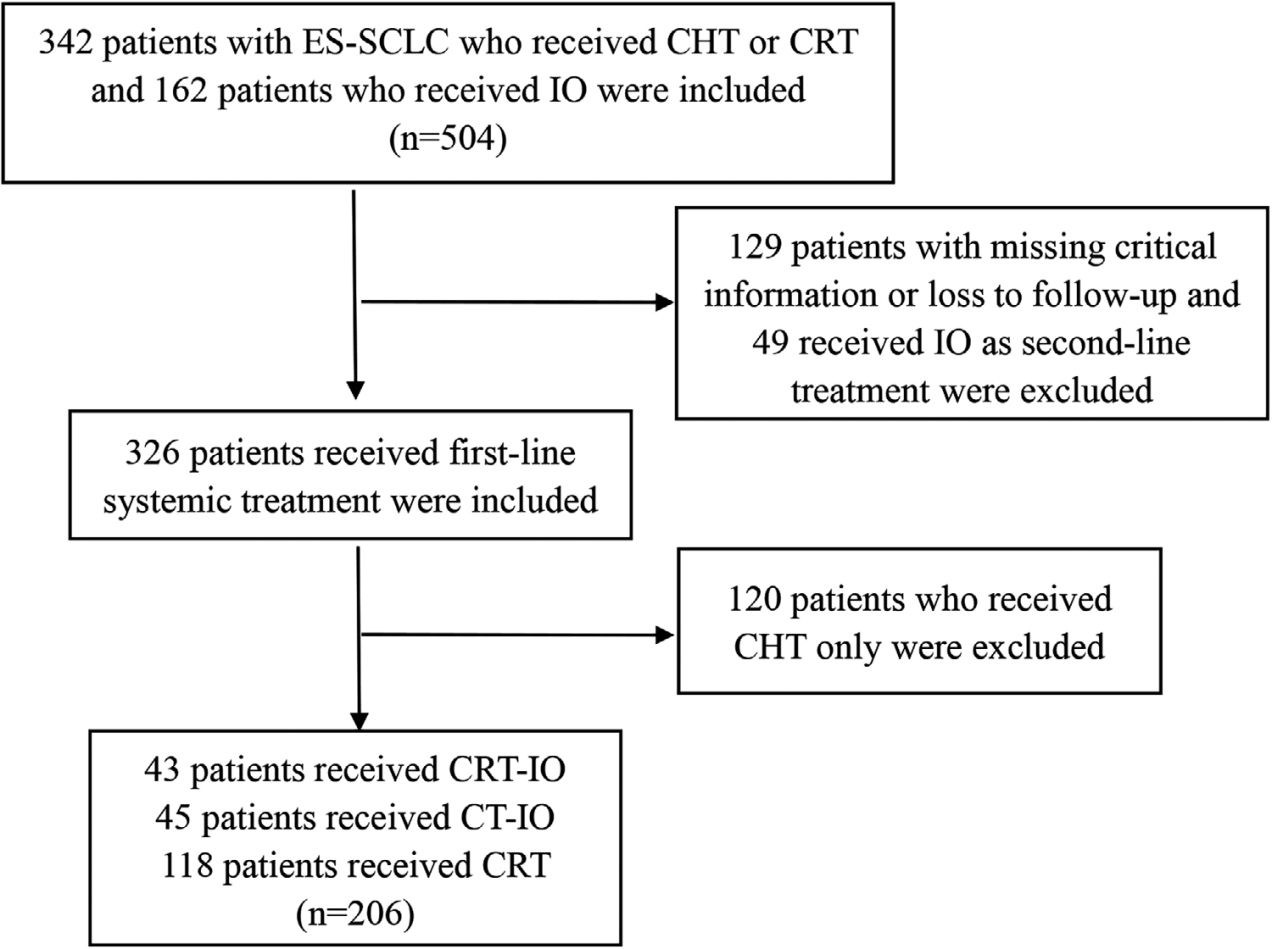
Patient selection flowchart. CHT, chemotherapy; CRT, chemoradiotherapy; CRT‐IO, chemoradioimmunotherapy; CT‐IO:chemoimmunotherapy ES‐SCLC, extensive‐stage small cell lung cancer; IO, immunotherapy.

In total, 342 patients diagnosed with ES‐SCLC who underwent CHT or CRT were included. An additional 162 patients underwent IO, resulting in a total of 504 patients. Following this, the exclusion criteria led to the removal of 129 cases due to incomplete data or loss of follow‐up. Additionally, 49 patients were excluded because they received IO and not first‐line treatment. Subsequently, 120 patients who underwent CHT alone were excluded. Data from 206 patients were included in this study (Figure 1). The patients were classified into three subgroups according to their individualized therapeutic strategies: 43 patients received CRT‐IO, 45 received CT‐IO, and 118 received CRT. Furthermore, we compared the CRT‐IO group with the CT‐IO (CRT‐IO vs. CT‐IO) and CRT (CRT‐IO vs. CRT) groups. The patients'clinical features are summarized in Table 1, with pairwise comparisons showing a balance in age, sex, and KPS score (all *P*>0.05).

**TABLE 1:** Patient Clinical Characteristics for ES‐SCLC between the CRT‐IO Group and the CT‐IO Group or the CRT Group.

Variables	CRT‐IO	CT‐IO	*p‐*value	CRT‐IO	CRT	*p‐*value
**Age, y**						
<60	19	18		19	52	
≥60	24	27	0.691	24	66	0.989
**Sex**						
Male	32	40		32	91	
Female	11	5	0.079	11	27	0.721
**Smoking index**						
≥400	23	32		23	51	
<400	20	13	0.088	20	67	0.247
**KPS score**						
>80	3	6		3	10	
≤80	40	39	0.485	40	108	1.000
**Weight loss**						
yes	7	5		7	15	
no	36	40	0.480	36	103	0.560
**Number of metastases**						
≤2	13	6		13	25	
>2	30	39	0.054	30	93	0.232
**Brain metastases**						
yes	30	25		30	79	
no	13	20	0.169	13	39	0.735
**Bone metastases**						
yes	16	19		16	39	
no	27	26	0.631	27	79	0.623
**Liver metastases**						
yes	16	24		16	29	
no	27	21	0.082	27	89	0.114

Abbreviations: CRT, chemoradiotherapy; CRT‐IO, chemoradioimmunotherapy; CT‐IO, chemoimmunotherapy; ES‐SCLC:extensive‐stage small cell lung cancer; KPS, Karnofsky Performance Status.

### 3.2 Survival data and analysis of risk factors

#### 3.2.1 CRT‐IO Group vs. CT‐IO Group

**FIGURE 2:**
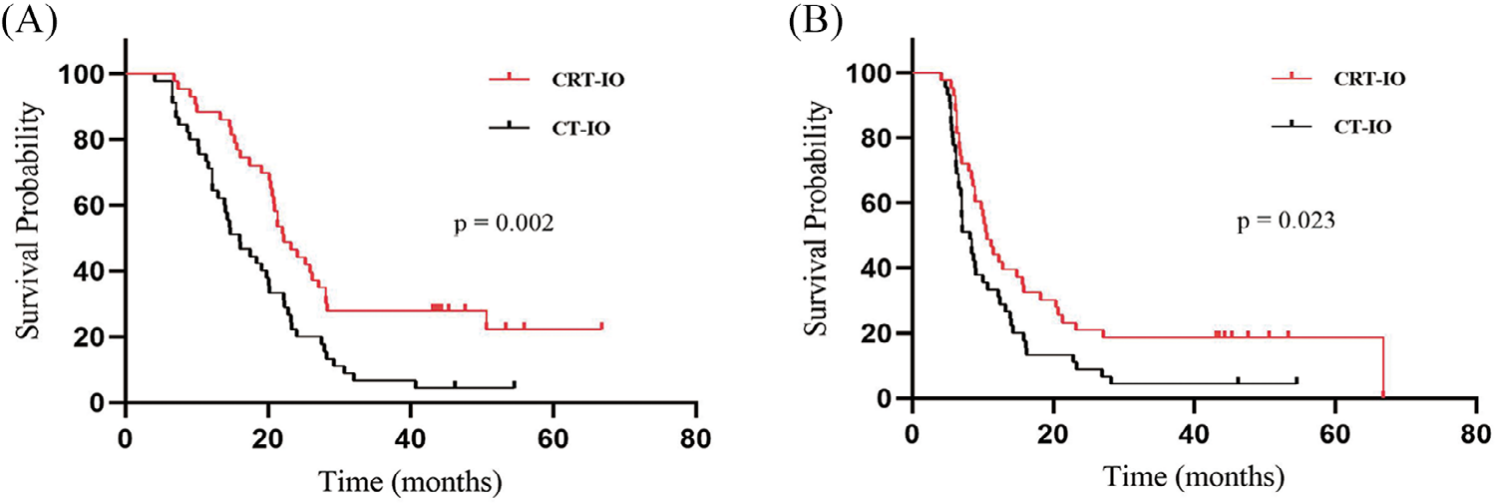
Kaplan–Meier survival curves of survival for patients with ES‐SCLC in the CRT‐IO and CT‐IO groups. (A) overall survival, (B) progression‐free survival, †ES‐SCLC: extensive‐stage small‐cell lung cancer; CRT‐IO: chemoradioimmunotherapy; CT‐IO: chemoimmunotherapy

The median follow‐up duration was 46.2 months. Our analytical results demonstrated that the median OS (mOS) was 22.2 months in the CRT‐IO group, which was longer than the 16.0 months observed in the CT‐IO group (*P* = 0.002). The CRT‐IO regimen was associated with a survival advantage. The regimen achieved a median PFS (mPFS) of 10.6 months, as opposed to 8.2 months in the CT‐IO cohort (*P* = 0.023, Figure 2).

**FIGURE 3:**
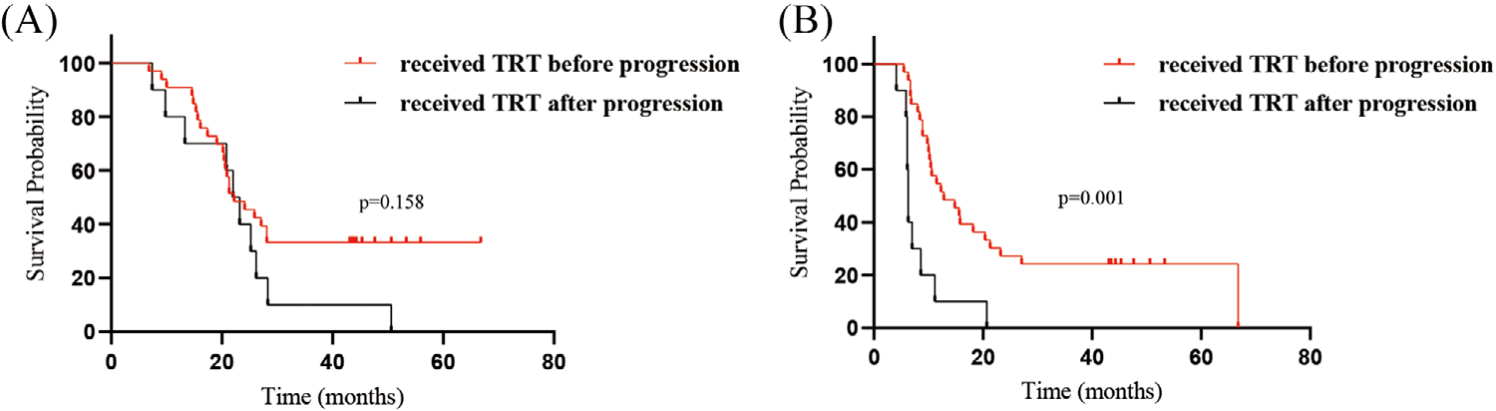
Kaplan–Meier survival curves of survival for patients with ES‐SCLC in the CRT‐IO group who received TRT before or after progression. (A) overall survival, (B) progression‐free survival, †ES‐SCLC: extensive‐stage small‐cell lung cancer; CRT‐IO: chemoradioimmunotherapy; TRT: thoracic radiotherapy

**FIGURE 4:**
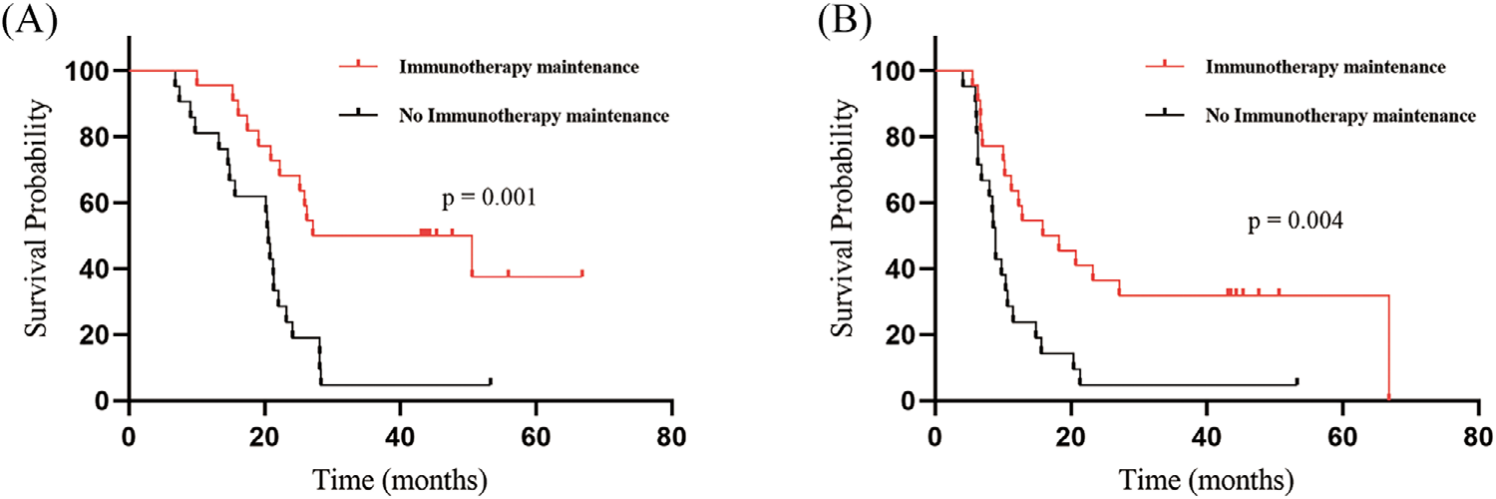
Kaplan‐Meier survival curves of survival for patients with ES‐SCLC in the CRT‐IO group who received IO‐maintenance or not. (A) overall survival, (B) progression‐free survival, †ES‐SCLC: extensive‐stage small‐cell lung cancer; CRT‐IO: chemoradioimmunotherapy; IO: immunotherapy

A detailed analysis was conducted on patients who underwent CRT‐IO; 33 of the 43 patients received TRT before disease progression, while the remaining 10 received TRT after disease progression. Superior mPFS was achieved in patients who received TRT before progression (12.8 months) compares to those who received TRT after progression (6.3 months, *P* = 0.001). Unfortunately, no significant difference in mOS (22.2 vs. 22.0 months, *P* = 0.158; Figure 3) was observed between these two groups. Additionally, 22 patients received IO maintenance therapy, whereas the remaining 21 did not. Patients receiving IO maintenance therapy exhibited improved mOS (27.1 vs. 20.6 months, *P* = 0.001). A similar survival benefit was observed for mPFS, with the IO maintenance cohort achieving 15.8 months vs. 8.9 months in nonrecipients (*P* = 0.004, Figure 4).

Univariate analysis of survival data revealed that a smoking index ≥400, KPS score ≤80, >2 metastases, bone metastases, liver metastases, and no TRT were related to shorter OS. Multivariate analysis revealed that independent predictors of OS included smoking index (*P* = 0.010), KPS score (*P*<0.010), bone metastases (*P* = 0.046), and number of metastases (*P* = 0.028) (Table 2).

**TABLE 2:** Univariate and multivariate analysis of OS in ES‐SCLC patients receiving IO.

	Univariate	Multivariate	
Variables	*p*‐value	HR (95%CI)	*p*‐value
Age, y (≥60 vs. <60)	0.593	‐	‐
Sex (Male vs. Female)	0.216	‐	‐
Smoking index (<400 vs. ≥400)	<0.001	2.01 (1.19,3.69)	0.010
KPS score (≤80 vs. >80)	<0.001	0.19 (0.09,0.42)	<0.001
Weight loss (yes vs. no)	0.542	‐	‐
Number of metastases (≤2 vs. >2)	0.001	2.36 (1.10,5.07)	0.028
Brain metastases (yes vs. no)	0.993	‐	‐
Bone metastases (yes vs. no)	0.001	1.63 (1.01,2.62)	0.046
Liver metastases (yes vs. no)	<0.001	1.40 (0.84,2.35)	0.200
TRT (yes vs. no)	0.009	0.67 (0.41,1.08)	0.097

Abbreviations: ES‐SCLC, extensive‐stage small cell lung cancer; IO, immunotherapy; KPS, Karnofsky Performance Status; OS, overall survival; TRT, thoracic therapy.

#### 3.2.2 CRT‐IO Group vs. CRT Group

**FIGURE 5:**
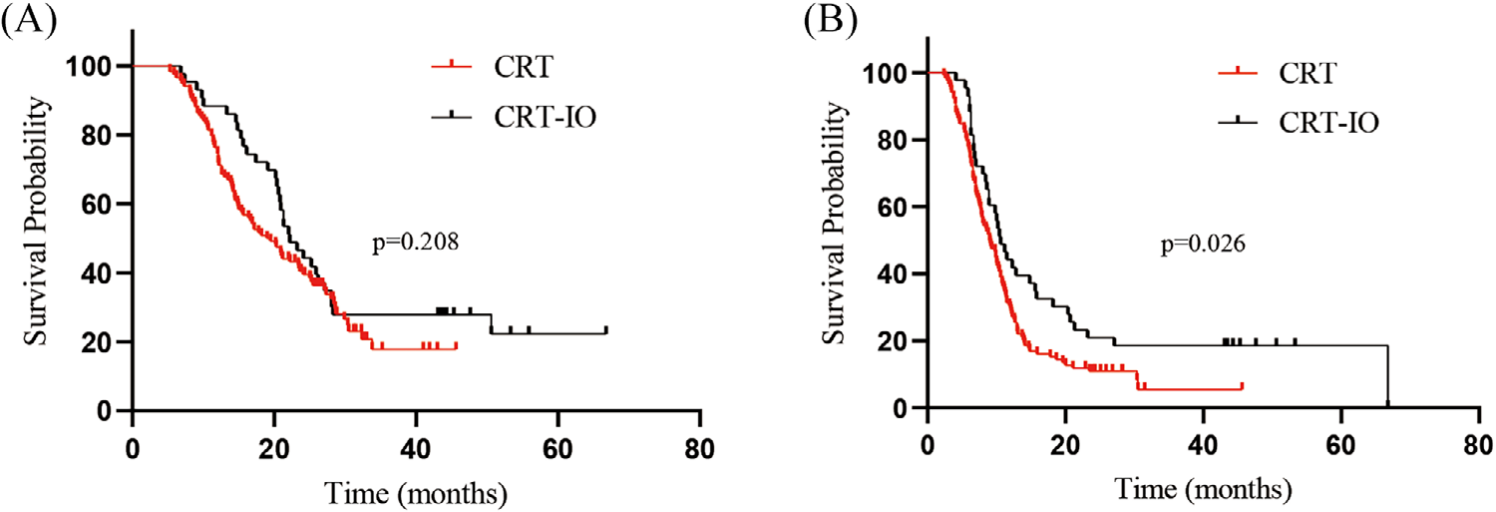
Kaplan–Meier survival curves of survival for patients with ES‐SCLC in the CRT‐IO and CRT groups. (A) overall survival, (B) progression‐free survival, †ES‐SCLC: extensive‐stage small‐cell lung cancer; CRT‐IO: chemoradioimmunotherapy; CRT: chemoradiotherapy

Patients assigned to the CRT‐IO regimen achieved an mOS of 22.2 months, whereas those in the CRT group achieved an mOS of 19.0 months (*P* = 0.208). The CRT‐IO group showed a numerical survival benefit compared to the CRT group, although the difference was not statistically significant. Moreover, mPFS was significantly better in the CRT‐IO group than in the CRT group (10.6 vs. 9.0 months; *P* = 0.026). Patients may benefit from the addition of IO, as shown in Figure 5.

Univariate analysis revealed that KPS score and number of metastases, bone metastases, and liver metastases were significant prognostic indicators of OS. In the multivariate Cox regression, prognostic factors associated with unfavorable OS outcomes included a KPS score ≤ 80 (*P* = 0.006), >2 metastases (*P* = 0.002), and liver metastases (*P* = 0.007). The results are summarized in Table 3.

**TABLE 3:** Univariate and multivariate analysis of OS in ES‐SCLC patients receiving TRT.

	Univariate	Multivariate	
Variables	*p*‐value	HR (95%CI)	*p*‐value
Age, y (≥60 vs. <60)	0.086	‐	‐
Sex (Male vs Female)	0.361	‐	‐
Smoking index (<400 vs. ≥400)	0.060	‐	‐
KPS score (≤80 vs. >80)	0.015	0.42 (0.23,0.78)	0.006
Weight loss (yes vs. no)	0.008	1.49 (0.89,2.49)	0.131
Number of metastases (≤2 vs. >2)	<0.001	2.46 (1.37,4.39)	0.002
Brain metastases (yes vs. no)	0.716	‐	‐
Bone metastases (yes vs. no)	0.001	1.38 (0.93,2.04)	0.111
Liver metastases (yes vs. no)	<0.001	1.76 (1.17,2.66)	0.007
IO (yes vs. no)	0.208	‐	‐

Abbreviations: ES‐SCLC, extensive‐stage small cell lung cancer; IO, immunotherapy; KPS, Karnofsky Performance Status; OS, overall survival; TRT, thoracic therapy.

### 3.3 Adverse events and tumor response

Regarding adverse events, the CRT‐IO group experienced a 27.9% (n = 12) incidence of grade 3/4 myelosuppression, while the CT‐IO group had a 22.2% (n = 10) incidence, and the CRT group had a grade 3/4 myelosuppression incidence of 27.1% (n = 32). No statistically significant differences were observed between the CRT‐IO and other two groups (*P* = 0.538 and *P* = 0.921, respectively).

Moreover, gastrointestinal reactions occurred more frequently in the CRT‐IO group than in the CT‐IO group (23.3% vs. 15.6%; *P* = 0.360). A similar trend was observed when compared with the CRT group, with rates of 23.3% vs. 18.6%; (*P* = 0.516), but with no statistically significant differences. Immune‐associated pneumonia occurred in three patients in each group. Tumor response was assessed using the objective remission rate (ORR). The CRT‐IO group demonstrated a superior ORR of 69.8% compared with the CT‐IO (68.9%, *P* = 0.929) and CRT (59.3%, *P* = 0.227) groups, although the between‐group differences were not significant.

## 4 DISCUSSION

ES‐SCLC remains a difficult‐to‐treat disease, with a poor survival prognosis over the last 30 years. Although the integration of IO into first‐line CHT improves survival, the results remain unsatisfactory. The IMpower133 trial showed that the addition of atezolizumab to EC regimens prolonged mPFS and mOS^4^. The CASPIAN study revealed that durvalumab added to EP had significant advantages regarding mOS compared with EP alone, and 33.9% of patients survived for >18 months, which increased the interest in evaluating the benefits and risks of CT‐IO.^5^ The latest results reported by ASCO in 2020 demonstrated prolonged survival. Moreover, nearly 1/4 of patients achieved a survival benefit exceeding 2 years, and nearly 1/5 achieved a survival benefit beyond 3 years, indicating the unique long‐tail effect of IO therapy.^6^ Positive outcomes from the CAPSTONE‐1 and ASTRUM‐05 trials support the use of adebrelimab or serplulimab with CHT as a first‐line treatment.^7^, ^8^ However, randomized trials have demonstrated the effects of IO compared to standardized treatment; IO combined with CHT can improve PFS by only approximately a month and OS by only two–three months. In addition, before the landmark Jeremic trial, TRT was never viewed as a consolidative part of treatment.^9^ This finding was recently confirmed by the CREST, RTOG 0937, and several retrospective studies,^10^, ^11^, ^12^, ^18^, ^19^, ^20^, ^21^, ^22^ which suggested that TRT, as a local treatment, could confer a survival advantage in CHT‐dominated management. Furthermore, evidence indicates that integrating TRT into IO can provide synergistic effects.^23^, ^24^ However, the clinical value of TRT combined with CT‐IO remains unclear. Therefore, this study aimed to compare the survival differences between CRT‐IO, CT‐IO, and CRT.

Our study indicated the superior efficacy of TRT, with a significantly longer mOS of 22.2 months in the CRT‐IO group than that of 16.0 months in the CT‐IO group (*P* = 0.002). Our results revealed that the survival time was better than that reported in the aforementioned randomized controlled trials.^5^, ^6^, ^7^, ^8^, ^9^ The possible reasons for this are as follows: only 88 patients received IO therapy in our analysis, which may have introduced bias owing to the insufficient sample size. Additionally, diverse treatments, such as anti‐angiogenic and anti‐CTLA‐4 therapies, may have been administered after disease progression. A significant improvement in mPFS was observed with CRT‐IO compared with CT‐IO (10.6 vs. 8.2 months, *P* = 0.023). Xie et al. and Peng et al. reached comparable conclusions.^25^, ^26^ These results are consistent with those of a retrospective analysis by Cai et al.^27^ Another multicenter retrospective analysis revealed better survival outcomes when CT‐IO was combined with TRT.^28^ However, a retrospective analysis by Li et al. reached a different conclusion, showing an extension in survival that was not statistically significant.^29^

Subsequent analysis demonstrated that the CRT‐IO group exhibited a statistically significant improvement in mPFS compared with that of the CRT group. The CRT‐IO group had an mPFS of 10.6 months, compared with 9.0 months in the CRT group (*P* = 0.026). Nevertheless, the mOS benefit was not statistically significant (*P* = 0.208), with the two groups achieving survival times of 22.2 and 19.0 months, respectively. The examination of 161 patients revealed a trend toward improved mOS. This finding indicates that CRT‐IO appears to prolong OS compared with CRT in patients with ES‐SCLC. In addition, compared with patients who received TRT after progression, those who received TRT before progression showed significantly better mPFS (12.8 vs. 6.3 months, *P* = 0.001). However, no survival benefit was observed in mOS (22.2 months vs. 22.0 months, *P* = 0.158). The radiation doses in the CRT‐IO group were not uniform. TRT was performed using different fractionation schemes, including urgent RT for superior vena cava syndrome, partly concurrent CHT, and premature termination of treatment for various reasons. Moreover, for patients receiving IO, the mOS (27.1 vs. 20.6 months, *P* = 0.001) and mPFS (15.8 vs. 8.9 months, *P* = 0.004) were better in those receiving IO maintenance than in nonrecipients. Overall, whether TRT combined with IO could provide a survival benefit requires further discussion.

Univariate and multivariate analyses suggested that CRT‐IO was associated with a lower mortality risk than CT‐IO and CRT; however, the differences were not statistically significant. Moreover, a slight advantage in ORR was observed in the CRT‐IO group compared to the CT‐IO group (69.8% vs. 68.9%, *P* = 0.929). Although there is insufficient evidence to support this hypothesis, CRT‐IO may be recommended for future studies. An analysis of the prognostic factors was subsequently performed to determine which patients would benefit the most from CRT‐IO. Compared with patients receiving CT‐IO, patients with excellent KPS scores and lower smoking indices may be more suitable for CRT‐IO and may benefit more from CRT‐IO than from CRT. CRT‐IO is recommended for these patients, as they may better tolerate high‐intensity therapy, resulting in better survival. The smoking index is an unfavorable factor that affects prognosis.^30^ Moreover, patients with ≥2 metastatic sites and liver metastases had poorer prognoses in our study. The effect of TRT in this high‐risk group remains under investigation. A study by Zhang et al.,^22^ focusing on patients with oligometastases, suggested that TRT may offer greater benefits to patients with fewer metastatic sites and yield superior outcomes compared to those without liver metastases. Another study by Zhu et al. reported that TRT significantly improved the survival of patients with oligometastases.^31^ Further studies are required to clarify the clinical value of TRT in high‐risk patients. IO acceptance was not an independent predictor of improved survival. The addition of IO to CRT in patients with ES‐SCLC resulted in a trend toward prolonged survival. Considering the economic and physical health of patients, CRT may be an option for patients with ES‐SCLC.

Survival gains from IO therapy are invariably linked to treatment‐related adverse events. Shaverdian et al. showed that TRT and concurrent pembrolizumab treatment exhibited good tolerability.^12^ Verma et al. demonstrated that the combination of IO and TRT was safe in the short term.^32^ A phase I clinical study by Welsh et al. demonstrated that TRT following CT‐IO had acceptable safety, and comparable findings were reported in a retrospective analysis by Li et al.^33^, ^34^ However, Perez et al. concluded early owing to negative results and a high incidence of immune‐related pneumonia.^35^ In summary, more focus should be placed on the incidence of adverse reactions while improving survival rates. Moreover, Higgins et al. suggested that the combination of TRT is reasonable for further exploration because the treatment effect of IO is modest.^36^ Our data indicated that the combination was tolerable; however, more attention should be paid to the gastrointestinal reactions of patients when receiving CRT‐IO. Currently, the safety of TRT after IO is mostly based on small sample sizes or retrospective clinical study data, which need to be tested in large‐scale randomized controlled trials.

Furthermore, although TMB and PD‐L1 have served as established biomarkers for patient selection in NSCLC,^37^, ^38^ specific markers for direct individualized therapy are currently lacking in patients with ES‐SCLC. Therefore, prognostic biomarkers require further exploration. The limitations of our study should be noted in addition to its retrospective nature. On the one hand, the radiation dose/fraction was not specified, and the treatment decisions were made by physicians based on their clinical experience and expertise, although this disadvantage could be reduced through discussion, at least to a certain extent. On the other hand, detailed treatment protocol data were unavailable because not all patients underwent the entire treatment course at our institution. Moreover, no biomarker analysis was performed, and long‐term toxicity was not evaluated in our study. However, the combination of TRT with different immuno‐oncological agents requires further evaluation.

In conclusion, whether TRT could become more important because of synergy and possibly enhance the antitumor immune response or whether TRT could be ignored in the era of IO based on the reduction in distant progression is a question worthy of consideration. Given the trend toward prolonged survival and higher ORR in the CRT‐IO group, we cautiously recommend that TRT is feasible in the IO era. Considering the economic factors and physical conditions, CRT may be a potential treatment option. Further trials are needed to explore novel combination therapies for patients with ES‐SCLC.

## CONFLICT OF INTEREST STATEMENT

No commercial or financial interests need to be declared.

## ETHICAL STATEMENT

Our study was approved by the Ethics Committee of the Shandong Cancer Hospital and Institute (approval number: SDTHEC202501068). Given the retrospective nature of our study, the requirement for informed consent was waived.

## Data Availability

The data that support the findings of this study are available on request from the corresponding author.

## References

[1] Siegel RL , Miller KD , Wagle NS , A Jemal . Cancer statistics. CA Cancer J Clin. 2023;73(1): 17–48. 36633525 10.3322/caac.21763

[2] Ganti AKP , Loo BW , Bassetti M , et al. Small cell lung cancer, version 2.2022, NCCN clinical practice guidelines in oncology. J Natl Compr Canc Netw. 2021;19:1441‐1464. 34902832 10.6004/jnccn.2021.0058 PMC10203822

[3] Su S , Zou JJ , Zeng YY , et al. Tumor mutational burden and genomic alterations in Chinese small cell lung cancer measured by whole‐exome sequencing. Biomed Res Int. 2019, 6096350. 31781628 10.1155/2019/6096350 PMC6874933

[4] Mansfield AS , Każarnowicz A , Karaseva N , et al. Safety and patient‐reported outcomes of atezolizumab, carboplatin, and etoposide in extensive‐stage small‐cell lung cancer (IMpower133): a randomized phase I/III trial. Ann Oncol. 2020;31(2):310‐317. 31959349 10.1016/j.annonc.2019.10.021

[5] Paz‐Ares L , Dvorkin M , Chen Y , et al. Durvalumab plus platinum‐etoposide versus platinum‐etoposide in first‐line treatment of extensive‐stage small‐cell lung cancer (CASPIAN): a randomised, controlled, open‐label, phase 3 trial. Lancet. 2019;394(10212):1929‐1939. 31590988 10.1016/S0140-6736(19)32222-6

[6] Paz‐Ares LG , Dvorkin M , Chen Y , et al. Durvalumab ± tremelimumab + platinum‐etoposide in first‐line extensivestage SCLC (ES‐SCLC): updated results from the phase III CASPIAN study. J Clin Oncol. 2020;38(15):9002.

[7] Wang J , Zhou C , Yao W , et al. Adebrelimab or placebo plus carboplatin and etoposide as first‐line treatment for extensive‐stage small‐cell lung cancer (CAPSTONE‐1): a multicentre, randomised, double‐blind, placebo‐controlled, phase 3 trial. Lancet Oncol. 2022;23(6):739‐747. 35576956 10.1016/S1470-2045(22)00224-8

[8] Cheng Y , Han L , Wu L , et al. Effect of first‐line serplulimab vs placebo added to chemotherapy on survival in patients with extensive‐stage small cell lung cancer: the ASTRUM‐005 randomized clinical trial. JAMA. 2022;328(14):1223‐1232. 36166026 10.1001/jama.2022.16464 PMC9516323

[9] Jeremic B , Shibamoto Y , Nikolic N , et al. Role of radiation therapy in the combined‐modality treatment of patients with extensive disease small cell lung cancer: a randomized study. J Clin Oncol. 1999;17(7):2092. 10561263 10.1200/JCO.1999.17.7.2092

[10] Slotman BJ , van Tinteren H , Praag JO , et al. Use of thoracic radiotherapy for extensive stage small‐cell lung cancer: a phase 3 randomised controlled trial. Lancet. 2015;385(9962):36‐42. 25230595 10.1016/S0140-6736(14)61085-0

[11] Gore EM , Hu C , Sun AY , et al. Randomized Phase II Study Comparing Prophylactic Cranial Irradiation Alone to Prophylactic Cranial Irradiation and Consolidative Extracranial Irradiation for Extensive‐Disease Small Cell Lung Cancer (ED SCLC): NRG Oncology RTOG 0937. J Thorac Oncol. 2017;12(10):1561‐1570. 28648948 10.1016/j.jtho.2017.06.015 PMC5610652

[12] Shaverdian N , Lisberg AE , Bornazyan K , et al. Previous radiotherapy and the clinical activity and toxicity of pembrolizumab in the treatment of non‐small‐cell lung cancer: a secondary analysis of the KEYNOTE‐001 phase 1 trial. Lancet Oncol. 2017;18(7):895‐903. 28551359 10.1016/S1470-2045(17)30380-7 PMC5538772

[13] Antonia SJ , Villegas A , Daniel D , et al. Overall survival with durvalumab after chemoradiotherapy in stage III NSCLC. N Engl J Med. 2018;379(24):2342‐2350. 10.1056/NEJMoa1809697 30280658

[14] Theelen W , Peulen NFH , Lalezari F , et al. Randomized phase II study of pembrolizumab after stereotactic body radiotherapy (SBRT) versus pembrolizumab alone in patients with advanced non‐small cell lung cancer: The PEMBRO‐RT study. J Clin Oncol. 2018;36(15_suppl):9023.

[15] Wu L , Cheng B , Sun X , et al. Induction immunochemotherapy followed by definitive chemoradiotherapy for unresectable locally advanced non‐small cell lung cancer: a multi‐institutional retrospective cohort study. MedComm. 2024;5(3):e501. 38434760 10.1002/mco2.501 PMC10908364

[16] Wu L , Zhang Z , Bai M. et al. Radiation combined with immune checkpoint inhibitors for unresectable locally advanced non‐small cell lung cancer: synergistic mechanisms, current state, challenges, and orientations. Cell Commun Signal. 2023;21(1):119. 37221584 10.1186/s12964-023-01139-8 PMC10207766

[17] Welsh JW , Heymach JV , Chen D , et al. Phase I trial of pembrolizumab and radiation therapy after induction chemotherapy for extensive‐stage small cell lung cancer. J Thorac Oncol. 2020;15(2):266‐273. 31605794 10.1016/j.jtho.2019.10.001

[18] Zhu H , Zhou Z , Wang Y , et al. Thoracic radiation therapy improves the overall survival of patients with extensive‐stage small cell lung cancer with distant metastasis. Cancer. 2011;117(23):5423‐5431. 21563176 10.1002/cncr.26206

[19] Giuliani ME , Atallah S , Sun A , et al. Clinical outcomes of extensive stage small cell lung carcinoma patients treated with consolidative thoracic radiotherapy. Clin Lung Cancer. 2011;12(6):375‐379. 21729647 10.1016/j.cllc.2011.03.028

[20] Wu C , Wang T , Wang J , et al. Effect of radiotherapy on the treatment of patients with extensive stage small cell lung cancer. Genet Mol Res. 2014;13(4):8577‐8585. 24615077 10.4238/2014.January.24.7

[21] Han J , Fu C , Li BS. Clinical outcomes of extensive‐stage small cell lung cancer patients treated with thoracic radiotherapy at different times and fractionations. Radiat Oncol. 2021;16(1):47. 33663551 10.1186/s13014-021-01773-x PMC7934361

[22] Zhang H , Deng L , Wang X , et al. Metastatic location of extensive stage small‐cell lung cancer: implications for thoracic radiation. J Cancer Res Clin Oncol. 2019;145(10):2605‐2612. 31410604 10.1007/s00432-019-03000-3 PMC11810317

[23] Dovedi SJ , Cheadle EJ , Popple AL , et al. Fractionated Radiation Therapy Stimulates Antitumor Immunity Mediated by Both Resident and Infiltrating Polyclonal T‐cell Populations when Combined with PD‐1 Blockade. Clin Cancer Res. 2017;23(18):5514‐5526. 28533222 10.1158/1078-0432.CCR-16-1673

[24] Herrera FG , Bourhis J , Coukos G. Radiotherapy combination opportunities leveraging immunity for the next oncology practice. CA Cancer J Clin. 2017;67(1):65‐85. 27570942 10.3322/caac.21358

[25] Xie Z , Liu J , Wu M , et al. Real‐world efficacy and safety of thoracic radiotherapy after first‐line chemo‐immunotherapy in extensive‐stage small‐cell lung cancer. J Clin Med. 2023;12(11):3828. 37298023 10.3390/jcm12113828 PMC10253710

[26] Peng J , Zhang L , Wang L , et al. Real‐world outcomes of PD‐L1 inhibitors combined with thoracic radiotherapy in the first‐line treatment of extensive stage small cell lung cancer. Radiat Oncol. 2023;18(1):111. 37403111 10.1186/s13014-023-02308-2 PMC10320989

[27] Cai Z , Gu X , Xie J , et al. Safety and efficacy of thoracic radiotherapy combined with chemo‐immunotherapy in patients with extensive‐stage small cell lung cancer: a multicenter retrospective analysis. Transl Lung Cancer Res. 2023;12(10):1987‐2000. 38025813 10.21037/tlcr-23-294 PMC10654438

[28] Yao Y , Li B , Song R , et al. Efficacy and safety of thoracic radiotherapy in extensive‐stage small‐cell lung cancer patients receiving first‐line immunotherapy plus chemotherapy: a propensity score matched multicentre retrospective analysis. Radiat Oncol. 2024;19(1):25. 38413988 10.1186/s13014-024-02420-x PMC10900720

[29] Li YY , Jing W , Jing XQ , et al. Role of consolidative thoracic radiation in extensive‐stage small‐cell lung cancer with first‐line chemoimmunotherapy: a retrospective study from a single cancer center. Discov Oncol. 2023;14(1):55. 37142872 10.1007/s12672-023-00666-7 PMC10160328

[30] Ou SHI , Ziogas A , Zell JA. Prognostic factors for survival in extensive stage small cell lung cancer (ED‐SCLC): the importance of smoking history, socioeconomic and marital statuses, and ethnicity. J Thorac Oncol. 2009;4(1):37‐43. 19096304 10.1097/JTO.0b013e31819140fb

[31] Zhu Z , Zheng Z , Chu X , et al. High‐dose thoracic radiotherapy improves the overall survival of oligo‐metastatic extensive‐stage small cell lung cancer [abstract]. J Thorac Oncol. 2022;17(9 Suppl):S146‐S147.

[32] Verma V , Cushman TR , Selek U , et al. Safety of combined immunotherapy and thoracic radiation therapy: analysis of 3 single‐institutional phase I/II trials. Int J Radiat Oncol Biol Phys. 2018;101(5):1141‐1148. 30012526 10.1016/j.ijrobp.2018.04.054

[33] Welsh JW , Heymach JV , Chen D , et al. Phase I trial of pembrolizumab and radiation therapy after induction chemotherapy for extensive‐stage small cell lung cancer. J Thorac Oncol. 2020;15(2):266‐273. 31605794 10.1016/j.jtho.2019.10.001

[34] Li L , Yang D , Min Y , et al. First‐line atezolizumab/durvalumab plus platinum‐etoposide combined with radiotherapy in extensive‐stage small‐cell lung cancer. BMC Cancer. 2023;23(1):318. 37024843 10.1186/s12885-023-10784-8 PMC10080806

[35] Perez BA , Kim S , Dilling TJ , et al. A prospective single arm phase I/II study: consolidative ipilimumab and nivolumab with thoracic radiotherapy after platinum based chemotherapy for patients with extensive stage small cell lung cancer [abstract]. Int J Radiat Oncol Biol Phys. 2019;105(3 Suppl):S36.

[36] Higgins KA , Slotman BJ. What is the role of consolidative thoracic radiotherapy in the era of chemo‐immunotherapy for extensive stage small cell lung cancer? J Thorac Dis. 2020;12(10):6308‐6310. 33209469 10.21037/jtd.2020.03.15 PMC7656365

[37] Reck M , Rodríguez‐Abreu D , Robinson AG , et al. Updated analysis of KEYNOTE‐024: pembrolizumab versus platinum‐based chemotherapy for advanced non‐small‐cell lung cancer with PD‐L1 tumor proportion score of 50% or greater. J Clin Oncol. 2019;37(7):537‐546. 30620668 10.1200/JCO.18.00149

[38] Spigel D , de Marinis F , Giaccone G , et al. IMpower110: interim overall survival analysis of a phase III study of atezolizumab vs platinum‐based chemotherapy as first‐line treatment in PD‐L1–selected NSCLC. Ann Oncol. 2019;30(5):915.

